# Enhancing Post-Expansion Chondrogenic Potential of Costochondral Cells in Self-Assembled Neocartilage

**DOI:** 10.1371/journal.pone.0056983

**Published:** 2013-02-21

**Authors:** Meghan K. Murphy, Daniel J. Huey, Andrew J. Reimer, Jerry C. Hu, Kyriacos A. Athanasiou

**Affiliations:** Department of Biomedical Engineering, University of California Davis, Davis, California, United States of America; University of Pittsburgh, United States of America

## Abstract

The insufficient healing capacity of articular cartilage necessitates mechanically functional biologic tissue replacements. Using cells to form biomimetic cartilage implants is met with the challenges of cell scarcity and donor site morbidity, requiring expanded cells that possess the ability to generate robust neocartilage. To address this, this study assesses the effects of expansion medium supplementation (bFGF, TFP, FBS) and self-assembled construct seeding density (2, 3, 4 million cells/5 mm dia. construct) on the ability of costochondral cells to generate biochemically and biomechanically robust neocartilage. Results show TFP (1 ng/mL TGF-β1, 5 ng/mL bFGF, 10 ng/mL PDGF) supplementation of serum-free chondrogenic expansion medium enhances the post-expansion chondrogenic potential of costochondral cells, evidenced by increased glycosaminoglycan content, decreased type I/II collagen ratio, and enhanced compressive properties. Low density (2 million cells/construct) enhances matrix synthesis and tensile and compressive mechanical properties. Combined, TFP and Low density interact to further enhance construct properties. That is, with TFP, Low density increases type II collagen content by over 100%, tensile stiffness by over 300%, and compressive moduli by over 140%, compared with High density. In conclusion, the interaction of TFP and Low density seeding enhances construct material properties, allowing for a mechanically functional, biomimetic cartilage to be formed using clinically relevant costochondral cells.

## Introduction

Articular cartilage injury manifests in joint pain and dysfunction as repair tissue is unable to recapitulate native biochemical and biomechanical properties [1]. The insufficient healing capacity of cartilage presents orthopedic challenges in a number of diarthrodial joints and affects both fibrous and hyaline cartilages. For instance, disorders of the temporomandibular joint (TMJ), including disc perforation and displacement, have been associated with progressive articular cartilage degeneration and the development of osteoarthritis. Importantly, disc displacement, termed internal derangement, has been observed in 70% of patients seeking treatment for symptoms of TMJ disorders (TMD), demonstrating a clear clinical need [2].

Current management strategies, including joint debridement, disc removal or replacement, and hemi/total joint replacement have demonstrated minimal long-term potential in reducing pain and improving joint function. Often revision surgery is required [3]–[5]. Autologous tissue grafting presents an attractive alternative solution due to the minimized concern for an immune-mediated fibrotic response and the potential for adaptive remodeling according to changes in load distribution. As such, costochondral grafting has been used in craniofacial reconstructions including mandibular and condylar replacements [6], [7]. However, complications include unpredictable or excessive graft growth or resorption, restricted or deviated range of motion, and fracture at the graft-host junction [8]. As an alternative, tissue engineering may present a more conservative approach to replace the damaged articular surface with functional, autologous neocartilage. However, tissue engineering efforts are met with the challenges of 1) identifying a source of healthy cells associated with minimal donor site morbidity and 2) developing conditions to expand these cells while maintaining their ability to generate biomechanically functional tissue. Addressing these challenges may lead to a therapeutic approach with long-term regenerative potential.

Costochondral cells can serve as a potential source for cartilage tissue engineering. Advantages of a costochondral cell source include ease of obtaining tissue biopsies for cell harvesting, minimal concern for a diseased cell population, and previously demonstrated success of these cells in producing cartilaginous tissue [9]–[11]. However, to develop a sufficiently large cell population for engineering tissue replacements, monolayer expansion must be employed. While anchorage-dependent monolayer expansion enhances the proliferative potential of chondrocytes, it has been demonstrated that chondrocytes lose their differentiated phenotype during expansion, indicated by a decrease in aggrecan synthesis and a switch from collagen type II to collagen type I synthesis [12], [13]. In an effort to mitigate these deleterious changes, previous work has demonstrated that growth factors may be used in monolayer culture to modulate chondrocyte dedifferentiation, proliferation, and phenotypic potential upon reintroduction to 3D culture [13]–[16]. Specifically, it has been demonstrated that a growth factor cocktail of transforming growth factor-β1 (TGF- β1), basic fibroblastic growth factor (bFGF), and platelet derived growth factor-bb (PDGF), termed TFP, enhances proliferation and post-expansion chondrogenic potential in osteoarthritic and non-diseased articular chondrocytes (ACs), as well as ear and nasal chondrocytes [10], [15], [17], [18]. Additionally, supplementation of expansion medium with bFGF alone has been shown to facilitate re-expression of the chondrocyte phenotype upon 3D construct redifferentiation [19]–[21]. As the effects of growth factor supplementation during the expansion of costochondral cells are under characterized, this work will determine which of three expansion medium formulations is the most beneficial to the post-expansion chondrogenic potential of costochondral cells.

Though not examined in costochondral cells, construct seeding density may offer a secondary means of decreasing the cell requirement for engineering efforts and an indirect means of manipulating cell-cell and cell-matrix interactions. Using articular chondrocytes, high density 3D systems promote the formation of cartilaginous matrix and phenotypic stability [22]. In a variety of tissue engineering systems involving alginate, agarose, and fibrin scaffolds, it has been demonstrated that chondrocyte phenotype, matrix deposition, and mechanical properties depend on initial seeding density [23]–[27]. Significantly, in a study of cell concentration in alginate beads, SOX9 expression and sulfated glycosaminoglycan (GAG) production were upregulated in porcine ACs encapsulated at low density, compared with high density [23]. In self-assembled primary bovine ACs biochemical and biomechanical properties increased toward an upper limit and an optimal seeding density of 3.75 million cells per 5 mm dia. construct was identified [28]. As a determinant of intercellular distances and the transport of soluble factors, seeding density is anticipated to affect cell-cell signaling and matrix secretion in self-assembled expanded costochondral cells [29].

To realize the potential of costochondral cells in scaffoldless self-assembly of engineered articular cartilage, this work explores the effects of 1) medium supplementation during monolayer expansion and 2) initial construct seeding density on the ability of cells to generate tissue rich in collagen and GAGs and possessing biomechanical properties approaching those of native TMJ cartilage. To address the hypotheses costochondral cells are expanded to third passage (P3) in 1) chondrogenic medium with bFGF supplementation, 2) chondrogenic medium with TFP supplementation, or 3) traditional expansion medium containing 10% fetal bovine serum (FBS). Cells are redifferentiated in aggregate culture for 1 wk, digested, and self-assembled at 1) Low density (2×10^6^ cells per 5 mm construct), 2) Intermediate density (3×10^6^ cells per 5 mm construct), or 3) High density (4×10^6^ cells per 5 mm construct). Regarding the expansion medium formulation, it is hypothesized that TFP and bFGF supplementation to serum-free chondrogenic medium will enhance the post-expansion chondrogenic potential of cells as compared to a traditional serum-containing expansion medium. Additionally, it is hypothesized that the cartilaginous quality of constructs formed with cells expanded in the presence of TFP will exceed that of cells expanded in the presence of bFGF. With regards to construct seeding density, it is hypothesized that a low seeding density will facilitate collagen and GAG matrix synthesis, yielding mechanically robust tissue with minimal cell use.

## Materials and Methods

### Chondrocyte Isolation and Expansion

Rib tissue from skeletally mature (approximately 3 mo) crossbred Hampshire porcine (UC Davis Dept. Animal Sciences Meat Laboratory) was obtained immediately after sacrifice. Costal cartilage of the four caudal asternal ribs was excised and perichondrium was resected from the surface of the cartilage. Remaining cartilage was minced into ∼ 1 mm pieces and digested in 0.2% collagenase type II (Worthington, Lakewood, NJ) in chemically defined chondrogenic culture medium (CHG) [Dulbecco’s modified Eagle medium (DMEM) with 4.5 g/L-glucose and GlutaMAX (Gibco, Grand Island, NY), 1% penicillin-streptomycin-fungizone (BD Biosciences, Bedford, MA), 1% ITS+ premix (BD Biosciences), 1% non-essential amino acids (Gibco), 100 nM dexamethasone, 50 µg/mL ascorbate-2-phosphaste, 40 µg/mL L-proline, and 100 µg/mL sodium pyruvate] containing 3% FBS for 18 hrs at 37°C. Cells were filtered, counted, and seeded in T-225 flasks at 2.5×10^4^ cells/cm^2^. Cells were expanded in one of three conditions: 1) CHG supplemented with 5 ng/ml bFGF alone (referred to as bFGF supplementation), 2) CHG supplemented with 1 ng/ml TGF-β1, 5 ng/ml bFGF, 10 ng/ml PDGF (referred to as TFP supplementation), or 3) traditional FBS-containing expansion medium [DMEM with 4.5 g/L-glucose and GlutaMAX containing 10% FBS, 1% penicillin-streptomycin-fungizone, 1% non-essential amino acids, 50 µg/mL ascorbate-2-phosphate (Sigma)] (referred to as FBS expansion). At 80–90% confluence, cells were passaged with 0.5% Trypsin-EDTA (Gibco) 3 times (P3). Cell proliferation was quantified at each passage as fold increase in cell number per time to confluence. Total proliferation over three passages is reported as the product of the fold increases at each passage.

### Costochondral Cell Redifferentiation

Cells were redifferentiated in aggregate culture as previously described [30]. Briefly, 10^6^ cells/mL CHG were cultured with 10 ng/ml TGF-β1 in 100 mm×20 mm petri dishes (BD Biosciences) coated with 1% molecular biology grade agarose (Fisher Scientific, Fair Lawn, NJ). Coating was used to prevent cell adhesion during culture. During the first 24 hours, suspensions were maintained on an orbital shaker with gentle shaking to generate uniform cell aggregates. Aggregates were maintained in static cultures for the remaining 6 days. Following 7 days of redifferentiation, aggregates were digested for 45 minutes in 0.5% Trypsin-EDTA, followed by 45 minutes in 0.2% collagenase type II on an orbital shaker at 37°C. Cells were filtered and counted before self-assembling.

### Construct Seeding and Culture

Cells were self-assembled into engineered constructs in non-adherent agarose wells as previously described [31]. Briefly, a sterile stainless steel mold consisting of 5 mm dia. cylindrical prongs was constructed to fit in 6 wells of a 48 well plate. To each of six wells, 800 µl of sterile molten 2% molecular biology grade agarose (Fisher Scientific) in phosphate buffered saline was added and the mold was set. Agarose solidified at room temperature and the mold was removed. Two changes of CHG medium were used to saturate the wells by the time of seeding. Cells were then seeded at three densities: Low (2×10^7^ cells/mL), Intermediate (3×10^7^ cells/mL), or High (4×10^7^ cells/mL). Specifically, constructs were seeded into the agarose wells in 100 µl aliquots each with 2×10^6^ (Low), 3×10^6^ (Intermediate), or 4×10^6^ (High) cells. After 8 days of culture in agarose wells, constructs were unconfined and placed in wells coated with a thin layer of agarose to prevent adhesion. Unconfinement ensured neotissue did not deform if outgrowing the agarose wells. Eight constructs were formed per group and constructs were cultured in CHG medium for a total of 4 wks from initial seeding.

### Histology

After 4 wks culture time, samples from each group were analyzed histologically. Samples were frozen in HistoPrep Frozen Tissue Embedding Media (Fisher Scientific) and cryosectioned to 14 µm. Sections were fixed in formalin and stained with Safranin-O/fast-green or picrosirius red for glycosaminoglycans or total collagen, respectively. Alizarin red staining was also performed to detect the presence of calcification. Immunohistological staining techniques were used to detect the presence and localization of types I and II collagen, as previously described [31]. Mouse anti-type I collagen monoclonal antibody at 1∶600 dilution (Accurate Chemical, Westbury NY) was used to detect type I collagen and rabbit anti-human type II collagen polyclonal antibody at 1∶200 dilution (Cedarlane Labs, Burlington, NC) was used to detect type II collagen.

### Biochemistry and ELISA

Samples from each construct were analyzed for biochemical content after 4 wks of construct culture. Samples were massed before and after 48 hrs lyophilization, and digested in 125 µg/mL papain (Sigma) in phosphate buffer (pH 6.5) containing 2 mM N-acetyl cysteine (Sigma) and 2 mM EDTA for 18 hrs at 60°C. Glycosaminoglycan content was quantified with Blyscan GAG assay (Bicolor, Westbury, NY), based on 1,9-dimethylmethyl blue binding. Total collagen was quantified after hydrolyzing samples with 2 N NaOH for 20 min at 110°C using a chloramine-T hydroxyproline assay with Sircol™ collagen standards (Bicolor).

Additionally, sandwich enzyme-linked immunosorbent assays (ELISA) were used to detect type I and II collagen. A second set of lyophilized samples from each group were suspended in 0.05 M acetic acid containing 0.5 M NaCl (pH 3.0) and digested in 10 mg/mL pepsin in 0.05 M acetic acid at 4°C with constant agitation for 4 days, followed by 1 mg/mL pancreatic elastase (Sigma) in 1× tris buffered saline at 4°C with constant agitation for 24 hrs. Briefly, capture antibodies (monoclonal mouse anti-porcine type I and II collagen, respectively, Chondrex Redmond, WA) were incubated overnight at 4°C, wells were blocked with 2% BSA, samples/standards were added and incubated overnight, and detection antibodies (biotinylated monoclonal mouse anti-porcine type I and II collagen, respectively, Chondrex) were added and incubated overnight. Streptavidin Peroxidase solution was added, followed by tetramethylbenzidine as the substrate for detection. HCl was used to stop the reaction and plates were read at 450 nm.

### Mechanical Testing

After 4 wks construct culture, constructs were tested in unconfined compression and uniaxial tension. Samples were cut through the dia. to establish a level testing surface. A 2 mm dia. punch was taken from the center of each construct for compressive testing. Using an Instron 5565, samples were preconditioned with 15 cycles of 5% compressive strain and then strained to 10% and 20% sequentially in a stress-relaxation test. As previously described, a Kelvin solid viscoelastic model was fit to the data to establish compressive material properties at each strain level: instantaneous modulus (E_i_), relaxation modulus (E_r_), and coefficient of viscosity (μ) [32]. Tensile testing was conducted using a Test Resources 840L. A second 2 mm punch was taken adjacent to the first to generate a dog-bone shape and paper tabs were used to established a consistent and paper tabs were used to established a consistent gauge length of 1.3 mm. Samples were elongated at a rate of 1% strain per second. Stress-strain curves were developed from the load-displacement curve. Young’s modulus (*E_Y_*) and ultimate tensile strength (UTS) were quantified.

### Statistics

To determine if seeding density and expansion media were significant factors, data were analyzed with a two-way analysis of variance (ANOVA). To determine a difference between treatment groups, data were analyzed with a one-way ANOVA. When indicated by the F-test (p<0.05), Tukey’s *post hoc* test was performed to determine specific effects of factor levels following the two-way ANOVA and specific effects of treatment combinations following the one-way ANOVA. Capital letters and Greek symbols illustrate significant differences (p<0.05) between the levels of the two factors (seeding density and expansion medium). Groups not connected by lower case letters are significantly different. Again, factor levels/groups not sharing a common character are considered significantly different.

## Results

### Monolayer Cell Morphology and Proliferation

**Figure 1:**
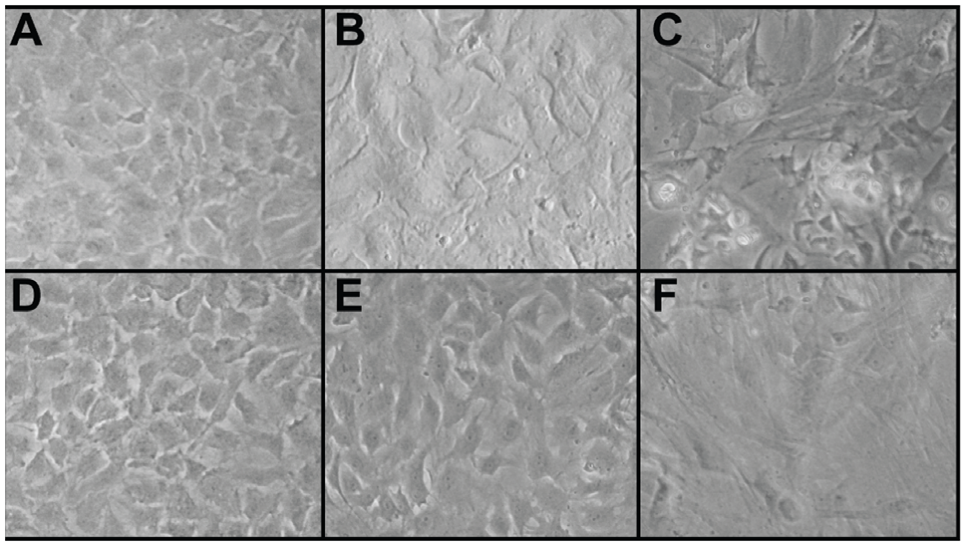
Cell Morphology in Monolayer. Morphology at confluence, prior to passaging at P1 (a–c) and P3 (d–f). Cells expanded in CHG with bFGF supplementation exhibited a rounded, cobblestone morphology upon confluence (a,d). Cells expanded in CHG with TFP (TGF-β1, bFGF, and PDGF) supplementation exhibited a polygonal, compact morphology upon confluence (b,e). Cells expanded in tranditional expansion medium containing 10% FBS exhibited a flattened, elongated cell morphology upon confluence (c,f).

Cell morphology in adherent monolayer culture is illustrated in Fig. 1. Images shown were collected immediately prior to first and third passages. Morphology and proliferation rates reflect cellular responses to growth factor treatments during monolayer expansion. Cells expanded in chondrogenic medium with TFP and bFGF supplementation demonstrated a similar morphology. In the presence of TFP, more developed cellular protrusions were apparent. Upon reaching confluence, both supplements yielded a densely packed, polygonal morphology. Cells expanded in traditional expansion medium containing 10% FBS demonstrated a much more flattened and elongated morphology. However, the morphology of cells expanded in the serum-supplemented medium was less homogeneous within passages. Cells expanded in bFGF and TFP showed a 3–4 fold increase in cell number upon reaching confluence while cells expanded in FBS showed a 2 fold increase in cell number upon reaching confluence. These differences demonstrate medium supplementation may be used to control the morphology and proliferative potential of costochondral cells in monolayer. Though expanded to P3, lower expansion numbers and proliferation rates resulting from the use of a serum-supplemented medium led to an insufficient cell population for self-assembling the High density group. As such, the FBS High density group has been omitted.

### Morphology, Histology, and Immunohistochemistry

**Figure 2:**
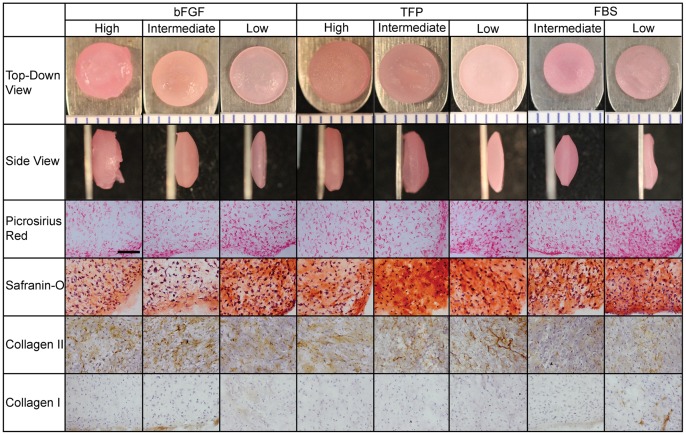
Construct Gross Morphology and Histology. Gross morphology and histology were imaged after 4 wks culture. All constructs stained positively for picrosirius red (collagen), Safranin-o (glycosaminoglycans), and type II collagen. More intense staining was apparent in Low density constructs. Type I collagen was detected in the periphery of constructs expanded with bFGF supplementation and minimally in constructs expanded in FBS-containing medium. Type I collagen was not detected in constructs expanded with TFP supplementation. Gross morphology ruler segments: 1 mm; histology scale bar: 100 µm.

After 4 wks culture in non-adherent agarose wells, costal chondrocytes generated robust neocartilage. Morphological differences among treatment groups are illustrated in Fig. 2. Construct morphology was similar across seeding densities in TFP and bFGF groups. Low seeding density yielded a stiff, bowl-shaped construct. Intermediate and High densities yielded a fluid-filled void region in the central portion of the construct. bFGF yielded larger voids, in some cases rupturing in the High density group. At all densities, FBS yielded voids within constructs. Hydration and diameter are shown in Table 1. Demonstrated by the two-way ANOVA, both Low seeding density and TFP expansion medium significantly reduced hydration, while TFP significantly increased construct diameter. For consistency, histology is reported for homogeneous tissue regions and is illustrated in Fig. 2. Picrosirius red and Safranin-O staining demonstrated the presence of collagen and GAG in all constructs. For all three medium supplementations, more intense matrix staining was observed in Low density constructs. Alizarin red staining for calcification was negative in all groups (data not shown). Immunohistochemistry demonstrated the presence of type II collagen in all groups, with more intense staining in Low density constructs. Type II collagen staining appeared diffuse in bFGF groups, as compared to intense staining of the collagen network apparent in TFP and FBS groups. Type I collagen staining was minimally detected in the FBS Low density group and in the periphery of constructs expanded with bFGF supplementation. Type I collagen was not detected in constructs expanded with TFP supplementation. IHC staining confirms the predominantly pericellular collagen distribution detected with picrosirius red staining. Despite differences in morphology, all constructs demonstrated the presence of collagen and GAG. Significantly, more intense GAG and type II collagen staining was apparent at Low density and in the presence of TFP supplementation.

**Table 1:** Construct Properties.

	% Water				Diameter (mm)		
Treatment	bFGF (A)	TFP (B)	FBS (A)	Treatment	bFGF (B)	TFP (A)	FBS (C)
High (α)	92.1±0.5^a^	91.2±0.6^ab^	not present	High (α)	5.7±0.3^bcd^	6.2±0.3^a^	not present
Intermed. (α)	92.0±0.7^a^	91.2±0.9^ab^	91.4±0.6^ab^	Intermed. (β)	5.7±0.2^bc^	5.8±0.2^bc^	5.3±0.3^d^
Low (β)	90.0±0.9^b^	88.7±0.7^c^	90.0±0.8^bc^	Low (αβ)	5.7±0.1^bcd^	5.8±0.1^b^	5.4±0.1^cd^

Construct hydration and diamater were quantified after 4 wks culture. Low seeding density significantly decreased hydration. TFP significantly decreased hydration and increased construct diameter. All data is presented mean ± s.d. Individual factor levels or groups not possessing a common letter are significantly different.

### Biochemical Content

**Figure 3:**
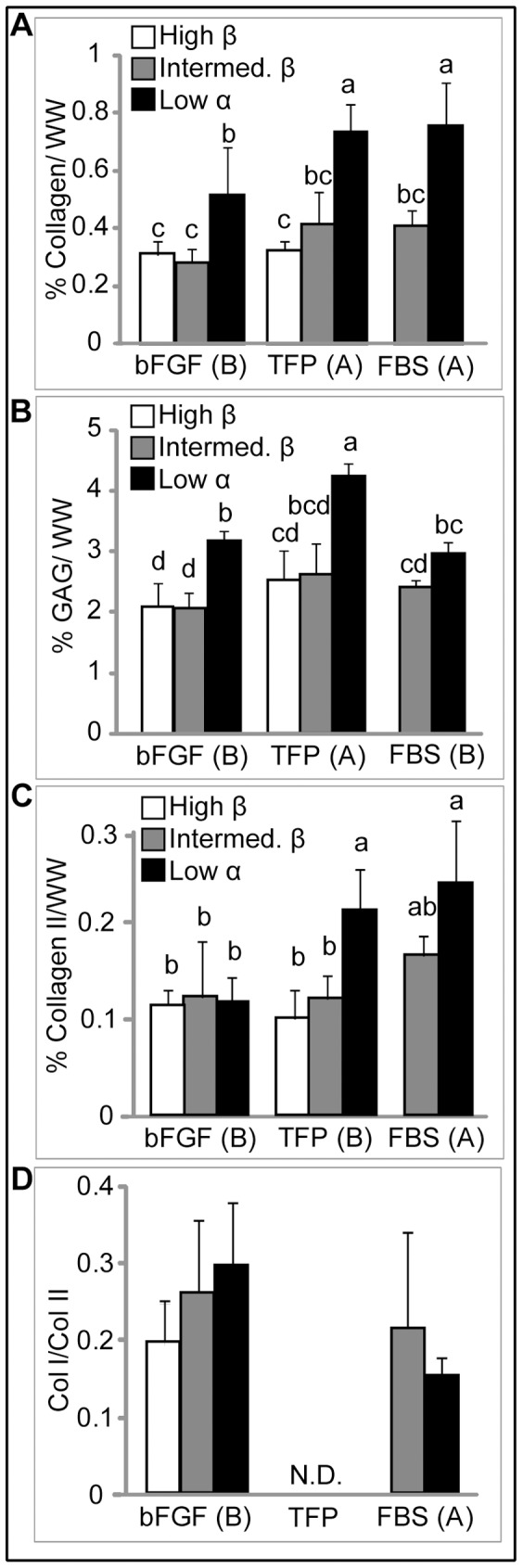
Biochemical Content of Neotissue. Biochemical content was assayed after 4 wks culture. Percent collagen (a), GAG (b), and collagen II (c) are reported per tissue wet weight. Collagen I/II ratio (d) is based on percent per tissue wet weight. Total collagen, GAG, and collagen II were significantly increased in Low density constructs. TFP expansion medium supplementation signficnatly increased GAG content while bFGF supplementation significantly increased the collagen I/II ratio. Type I collagen was below the limit of detection for constructs expanded with TFP supplementation (N.D.: not detected). All data is presented mean ± s.d. Individual factor levels or groups not possessing a common letter are significantly different.

Biochemical construct composition is reflected in Fig. 3, including total collagen, GAG, and collagen II percent by wet weight, as well as the type I/II collagen ratio. Additionally, Table 2 reflects biochemical content of constructs normalized to DNA content. A two-way ANOVA detected that both seeding density and expansion medium were significant factors in the matrix components assayed. Additionally, there was a significant interaction between these two factors for GAG (p = 0.0005) and type II collagen (p = 0.02). Total collagen and GAG content were significantly greater at Low seeding density. TFP and FBS resulted in significantly greater collagen content, compared with bFGF. Detected by a one-way ANOVA, collagen content was greatest in TFP and FBS Low density groups. TFP significantly increased GAG content over bFGF and FBS, independent of seeding density. Greatest GAG content was detected in the TFP Low density group. Significantly greater type II collagen was detected in Low density constructs, independent of medium supplementation. Type I collagen was detected only in bFGF and FBS groups, with no significant differences between groups. In the TFP groups, the type I/II collagen ratio is not reported because type I collagen was below the limit of detection. These results parallel those observed with histology and demonstrate enhanced chondrogenic potential of costochondral cells expanded in chondrogenic medium with TFP supplementation and self-assembled at Low density.

**Table 2:** Neotissue Biochemical Content Normalized to DNA.

	Collagen/DNA (ng/ng)				GAG/DNA (ng/ng)		
Treatment	bFGF (B)	TFP (A)	FBS (B)	Treatment	bFGF (B)	TFP (A)	FBS (C)
High (γ)	3.7±0.7^d^	4.9±0.7^cd^	not present	High (γ)	24.0±1.4^d^	38.2±8.7^b^	not present
Intermed. (β)	3.9±0.6^d^	7.2±1.3^ab^	4.8±1.3^cd^	Intermed. (β)	28.6±29^cd^	51.8±3.7^a^	25.7±0.2^cd^
Low (α)	6.4±2.4^bc^	9.3±0.7^a^	8.4±1.6^ab^	Low (α)	41.6±0.6^b^	56.0±2.6^a^	33.3±3.6^bc^

Biochemical content of neotissue was quantified after 4 wks culture. Values are normalized to DNA content. TFP supplementation significantly increased collagen/DNA and GAG/DNA compared with bFGF supplementation and FBS containing expansion medium. Additionally, decreasing seeding density significantly increased collagen and GAG content. All data is presented mean ± s.d. Individual factor levels or groups not possessing a common letter are significantly different.

### Mechanical Properties

**Figure 4:**
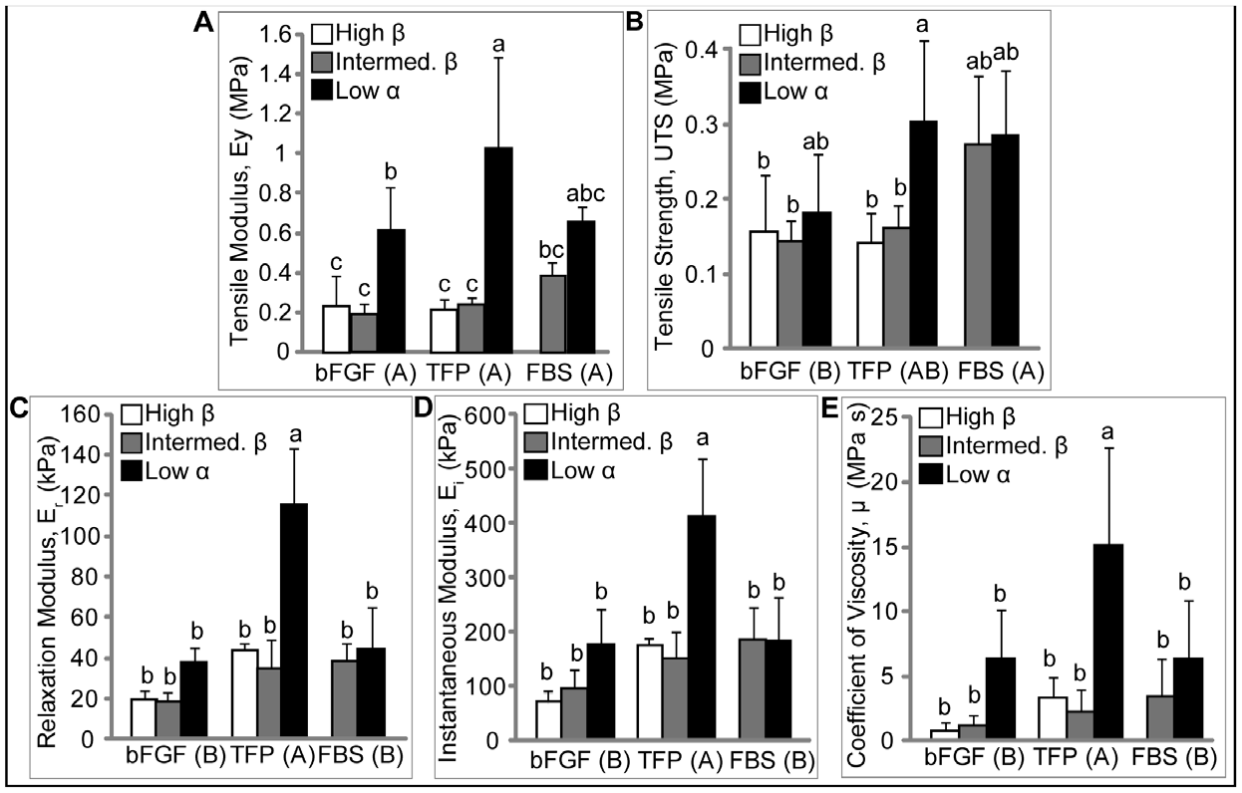
Mechanical Properties of Neotissue. Mechanical properties were assayed after 4 wks culture. Tensile modulus, E_y_, (a), ultimate tensile strength, UTS, (b), compressive relaxation, E_r_, (c) and instantaneous E_i_, (d) moduli, and coefficient of viscosity, μ, (e) are reported. Compressive properties are reported for 20% strain. Low seeding density significantly increased all mechanical properties assayed. TFP significantly increased compressive properties. All data is presented mean ± s.d. Individual factor levels or groups not possessing a common letter are significantly different.

Mechanical properties are reflected in Fig. 4. Detected by a two-way ANOVA, both seeding density and expansion medium were significant factors in biomechanical properties characterized. There was a significant interaction between these factors for tensile modulus (p = 0.02), compressive relaxation modulus (p<0.0001), instantaneous modulus (p<0.0001), and coefficient of viscosity (p = 0.03). At 20% compressive strain, Low seeding density significantly increased the coefficient of viscosity, and relaxation and instantaneous moduli. Additionally, TFP significantly increased the coefficient of viscosity, and relaxation and instantaneous moduli. Similar trends were observed at 10% strain. In tension, Low seeding density resulted in significantly stiffer and stronger constructs. Medium supplementation was not a significant factor in stiffness. Overall, Low density TFP constructs demonstrated significantly greater compressive properties and trended stiffer and stronger in tension, compared with all other groups. The mechanical properties detected here reflect the biochemical and histological results; TFP supplementation and Low seeding density yielded biochemically and mechanically robust neocartilage.

## Discussion

Degeneration in the articulating surfaces of diarthrodial joints and the poor outcome of current standards of care, especially in the case of TMD, have established a need for regenerative approaches with long-term potential. Engineering autologous tissue replacements presents an attractive solution. However, tissue engineering is met with the challenge of identifying a cell source obtained with minimal donor site morbidity and capable of generating biomechanically functional tissue. To address these challenges, costal cartilage was selected as a promising source of chondrocytes since it is unaffected by pathologies of diarthrodial joints. Costochondral grafts are currently in use by oral and maxillofacial surgeons and resection is associated with minimal donor site morbidity. This work explored the effects of chondrogenic expansion medium additives and construct seeding density on the ability of expanded costochondral cells to generate biochemically and biomechanically robust neocartilage. The central hypotheses of this study were that TFP expansion medium supplementation would best enhance the chondrogenic potential of expanded costochondral cells and that a lower initial construct seeding density would facilitate matrix synthesis, yielding mechanically robust tissue with minimized cell use. These hypotheses were proven as the TFP-supplemented expansion medium significantly increased construct GAG content and compressive properties and decreased the type I/II collagen ratio. Additionally, Low construct seeding density significantly increased collagen and GAG content, as well as tensile and compressive mechanical properties. Methodologies employed in this study allowed the formation of robust cartilage representing the spectrum of articular cartilage that one encounters in the TMJ, ranging from the fibrocartilage of the TMJ disc to the hyaline cartilage of the mandibular condyle.

Excitingly, this study demonstrates the post-expansion chondrogenic effects of TFP in serum-free medium. TFP supplementation to expansion medium has been shown to enhance the post-expansion chondrogenic potential of chondrocytes in a number of mammalian cell sources, and this work demonstrates this result in porcine costochondral cells. In human knee chondrocytes, post-expansion redifferentiation in 3D pellets was enhanced when cells were expanded to P2 in serum-containing medium supplemented with TFP, demonstrated by 2.4 fold increase in proteoglycan content and 2,500 fold increase in type II collagen mRNA expression [33]. Additionally, in assessing the effects of TFP with regard to donor-age, TFP-supplementation led to at most a 3.2 fold increase in GAG content for donors less than 40 yrs, and GAG content was correlated with type II collagen mRNA expression (r = +0.685) [17]. In the current study, the 27% increase in GAG content and 23% increase in type II collagen content in the presence of TFP supplementation, compared with bFGF supplementation, agrees with these past investigations. However, in a previous study of human non-load bearing cartilages, TFP had no effect on GAG content, type II collagen mRNA expression, or type II collagen staining of costochondral cell pellets, despite its chondrogenic benefit to ear and nasal chondrocytes [10]. Two fundamental differences exist between this work and ours: TFP was used to supplement traditional FBS containing expansion medium, rather than chondrogenic medium and a pellet based redifferentiation system was employed, rather than our aggregate system. The work reported here shows that, in the absence of serum and using an aggregate redifferentiation system, TFP significantly increases GAG content and compressive moduli, and decreases type I/II collagen ratio. Thus, it is suggested that TFP supplementation is more effective in enhancing the post-expansion chondrogenic potential of costochondral cells if used to supplement chondrogenic, serum-free medium following aggregate culture. A prior study has demonstrated that the aggregate redifferentiation system employed in this study is highly effective at promoting chondrogenesis as evidenced by its ability to increase the GAG content and type II/I collagen ratio of expanded leporine chondrocyte constructs compared to primary chondrocyte constructs [30]. As such, significantly, this aggregate system may be contributing to the increased chondrogenesis not previously observed by Tay et al [10]. It was demonstrated that TFP supplementation of chondrogenic expansion medium, followed by aggregate redifferentiation, enhanced the cartilaginous matrix production of self-assembled costochondral cells.

TFP may enhance the redifferentiation of chondrocytes by downregulating RhoA signaling in adherent monolayer. Specifically, TFP may enhance the redifferentiation of chondrocytes when introduced to 3D culture by holding the cells in a minimally dedifferentiated, chondrocyte-primed state during monolayer expansion. It has been shown that TFP enhances dedifferentiation of human ACs in expansion, yet it subsequently enhances the cells’ capacity to redifferentiate to a chondrocyte phenotype in 3D [33]. It is possible that downregulation of the RhoA/ROCK signaling pathway during expansion with TFP supplementation may be a mediator in the enhanced redifferentiation potential observed previously in human ACs and in porcine costochondral cells here. In the current work, the presence of TFP resulted in a tightly packed cobblestone cell morphology compared with the flattened, and elongated morphology demonstrated by cells expanded in the presence of FBS. Through actin organization and SOX9 expression, the RhoA/ROCK signaling pathway has been found to modulate this morphologic change and directly affect phenotypic alterations associated with dedifferentiation [34], [35]. Overexpression of RhoA in the chondrogenic cell line ATDC5 was associated with cell spreading and stress fiber formation, and decreased SOX9 expression and GAG content. In our study, this cell shape is similar to the cellular morphology observed when FBS supplementation was provided [34]. Conversely, inhibition of ROCK signaling by Y27632 induced rounded cell morphology with minimal stress-fiber formation in monolayer, and in micromass culture, increased GAG synthesis and elevated SOX9 expression [34]. In the current study, cells expanded in TFP maintained a polygonal morphology in monolayer and matrix synthesis in 3D reflective of RhoA/ROCK downregulation. As such, the chondrocytes expanded in the presence of TFP may be held in a minimally dedifferentiated, proliferative phenotype, preventing the development of a type I collagen secreting fibroblastic phenotype that is associated with RhoA upregulation. The mechanism of action of TFP supplementation in chondrocyte expansion requires further work but it is clear in this work and in previous efforts that TFP supplementation enhances chondrogenic potential of expanded chondrocytes upon reintroduction to a 3D environment.

The hypothesis that chondrogenic medium with bFGF supplementation would enhance the ability of cells to form robust neocartilage, compared with traditional expansion medium with FBS, was disproven. The ability of chondrocytes to produce cartilaginous matrix following expansion has been shown to decrease as expansion number increases [13], [36]. In this work, passage number was held constant. However, two primary differences in monolayer phenotype led to variability in the number of cell doublings: 1) cell morphology demonstrated in Fig. 1, and 2) proliferation rate. Cells with FBS supplementation expanded slowly (10 fold increase in cell number in 19 days) and were flattened and larger. Cells expanded with TFP and bFGF supplementation demonstrated a tightly packed, smaller morphology and proliferated substantially faster (55 fold increase over 13 days with TFP and 42 fold increase over 14 days with bFGF alone). Despite passage number being held constant, the variability in cell shape and size led to less than half as many cells per surface area at confluence with serum-containing expansion medium, compared to TFP and bFGF supplemented chondrogenic medium. This resulted in an insufficient cell number for self-assembling the FBS High density group and further reduces the translational potential of this treatment condition. However, the fewer number of cell doublings with serum-containing medium may be a factor in the relatively superior cartilage-forming ability of these cells, compared to those expanded in chondrogenic medium with bFGF alone. Although, no differences were observed in GAG content with respect to these two conditions, FBS led to a 57% increase in total collagen, a 72% increase in collagen II, and over 50% increase in compressive and tensile properties, compared to bFGF. The decrease in chondrogenic capacity of cells with increasing expansion number is a likely cause in the relatively lower cartilaginous quality of constructs produced by cells expanded in chondrogenic medium supplemented with bFGF alone. Conversely, cells expanded with TFP demonstrated faster and greater cell doubling compared with those expanded with bFGF alone, and importantly, generated neocartilage with the greatest GAG and type II collagen content, and mechanical properties. This result further emphasizes the potent chondrogenic effect of TFP supplementation.

Seeding density is a key determinant in the quality of engineered cartilage and, historically, benefits have been observed in high densities [24]–[27], [37]. Seeding density affects engineered cartilage morphology, matrix accumulation and organization, and mechanical properties in the presence of various scaffolding biomaterials and in their absence. Increasing density has been associated with increased matrix synthesis and mechanical integrity [25], [38]. In chondrocyte seeded-polyglycolic acid scaffolds, higher seeding density (10×10^6^ cells per 10 mm dia. × 5 mm thickness disc, corresponding to approximately 25×10^6^ cells/mL) yielded significantly higher GAG and collagen content, when compared with lower seeding densities (2 or 4×10^6^ cells per scaffold) [38]. Similarly in agarose hydrogels, 60×10^6^ cells/mL increased mechanical properties and biochemical content compared with 20×10^6^ cells/mL [25]. In articular chondrocytes seeded on PGA scaffolds, higher seeding densities (20×10^6^ cells/mL and 100×10^6^ cells/mL) showed a higher percentage of cartilage formation when compared with low density (2×10^6^ cells/mL) [39]. A similar observation has been made in costal chondrocytes seeded on poly (glycerol sebacate); 50 and 100 million cells/mL increased collagen and GAG content compared with 25 million cells/mL [37]. In contrast to the previously demonstrated benefits of increasing density, the work presented here demonstrates benefit of a lower density. This may be explained by the final cell density of Low density constructs engineered in this work (125–150×10^6^ cells/mL) being on par with high density conditions in previous studies. As such, the notable biochemical content (3.5% GAG/WW) and mechanical properties (0.8 MPa tensile modulus, 66 kPa relaxation modulus, and 259 kPa instantaneous modulus) of Low density constructs, on average, are in agreement with previous efforts. Additionally, this work employs a scaffoldless, self-assembly system in comparison to the scaffold-based systems in which seeding density has been more thoroughly investigated. It must be emphasized that the specific seeding density and growth factor combination demonstrated to be most favorable in this self-assembly system may not be most ideal in 3D polymeric scaffold systems. Further work is needed to standardize the most beneficial seeding density and growth factor cocktail across biomaterials in costochondral cell cartilage formation. The work presented in this study affirms the previously demonstrated benefit of high seeding densities in engineering cartilage.

Beyond an upper limit of construct seeding density, the cartilaginous quality of neocartilage has been shown to plateau or fall [28], [40], paralleling trends observed in this work. In an alginate bead system, GAG accumulation was shown to peak in the range of 10×10^6^ cells/mL and decrease as cell density increased further. It was suggested that a balance must be struck to obtain a sufficient number of cells for robust matrix synthesis while not limiting metabolic activity or inducing apoptosis due to insufficient nutrient supply or waste removal [40]. Previous work with self-assembled primary bovine articular chondrocytes observed a plateau in salient construct properties as seeding density increased to 3.75 million cells per 5 mm dia. construct [28]. As such, there were no concerns for insufficient nutrient delivery or diffusional limitations when selecting the seeding densities employed in this work. However, fluid filled voids were observed in Intermediate and High density groups when expanded with TFP and bFGF supplementation, and at all densities when expanded with FBS. Construct biochemical and biomechanical properties were characterized for homogeneous tissue regions indicating the increase in construct properties at Low density was not influenced by these void areas. Nonetheless, the source of this void region remains unclear and requires further exploration. An initial hypothesis regarding the development of the void region is that inhomogeneous cell populations that result from monolayer expansion migrate and segregate, as postulated by the differential adhesion hypothesis [41]. This study demonstrated an upper limit in the mechanical and biochemical properties of engineered constructs, paralleling that observed previously in similar systems.

TFP and Low seeding density demonstrate an interaction that significantly enhances the salient properties of engineered cartilage. As described, Low seeding density increased matrix synthesis and mechanical properties of resulting neocartilage. TFP expansion medium supplementation enhanced the post-expansion cartilage forming capacity of costochondral cells. Applied together, these stimuli interact yielding neotissue with properties greater than that would be expected based on the two stimuli acting independently. Using a two-way ANOVA, the interaction term between the two factors of this study was significant for compressive moduli, coefficient of viscosity, and tensile stiffness. Biochemically, the interaction term was significant for total collagen, type II collagen, and GAG content. The presence of a significant interaction term demonstrates that the effects of each medium formulation depend on the seeding density used. Specifically, in the presence of TFP, Low density led to over 100% increase in type II collagen content, over 300% increase in tensile stiffness, and in compression, over 140% increase in both instantaneous and relaxation moduli, compared with High density. Combined TFP and Low seeding density interact in a way that is most beneficial to engineered costochondral cell neocartilage and resulted in constructs with near native properties.

Neocartilage engineered in this work achieved properties in range of native tissue values, including GAG content and compressive properties of articular cartilage. Specifically, the biochemical content and compressive properties of Low density, TFP-supplemented constructs parallel those of TMJ condylar cartilage and hyaline articular cartilage. Micro-indentation creep testing of native porcine TMJ condylar cartilage has demonstrated apparent aggregate moduli on the order of 75 kPa in its stiffest regions [42]. Compressive properties were tested in stress-relaxation herein and the Low density TFP group demonstrated a relaxation modulus over 100 kPa, achieving the range of native tissue values. Condylar cartilage has demonstrated anisotropic tensile properties, with Young’s modulus measured as 6.6 MPa mediolaterally and 9.0 MPa anteroposteriorly. Low density TFP constructs achieved a tensile modulus over 1 MPa. Though below native tissue values, significant improvements were achieved with this group toward native values. Similar to hyaline articular cartilage, the mature and hypertrophic zones of TMJ condylar cartilage possess a matrix rich in type II collagen and aggrecan [43]. With 4% GAG/ww (30.7±1.8% GAG/dw) and a collagen matrix composed almost exclusively of type II collagen, the biochemical content of the TFP Low density constructs parallels that of hyaline articular cartilage (15–30% proteoglycan by dry weight and predominantly type II collagen, >50% by dry weight) [38]. Conversely, the lower GAG content and increased type I/II collagen ratio of constructs expanded with FBS and bFGF is more reflective of the biochemical composition of the fibrocartilages of the TMJ (∼5% proteoglycan by dry weight, predominantly type I collagen). Significantly, after 4 wks of culture, the final cellular density of Low seeding-density constructs was in the range of 125–150×10^6^ cells/mL. This falls within the range of native articular cartilage cellularity: 50×10^6^ cells/mL to 310×10^6^ cells/mL depending on age, species, and depth, indicating intercellular distances were appropriate for chondrocyte cell signaling [44]. Approximation of native tissue cell density may have promoted matrix production and development of physiologically-relevant mechanical properties. Cartilage constructs engineered in this work achieved biochemical and mechanical properties within the range of native TMJ articular cartilage properties, with the exception of collagen content.

Frequently in tissue engineering, total collagen content detected in constructs is a fraction of native values despite the presence of near native mechanical properties, as observed in this work. By wet weight, native articular cartilage is composed of approximately 10–30% collagen. In contrast, engineered cartilage from a variety of groups has demonstrated collagen content between 0.3–4%/ww, yet tensile moduli range from 100 kPa to less than 10 MPa [19], [45]–[47]. Considering the structure-function relationship of cartilage, this discrepancy has remained puzzling. The presence of collagen in biologic tissues is readily determined through a colorimetric reaction involving Ehrlich’s solution and oxidized soluble hydroxyproline [48]. The assay requires first solubilization of hydroxyproline by alkaline hydrolysis and, in a neutral solution, oxidation by chloramine T of free hydroxyproline to produce a pyrrole [49]. A collagen or hydroxyproline standard is required to relate the color change to a known collagen quantity and in this work bovine skin collagen (Sircol) was used. In native tissue, the frequency of hydroxyproline (Hyp) residues in collagen molecules ranges from 98–103 Hyp residues/1000 residues and the frequency of proline (Pro) ranges from 65–124 Pro residues/1000 residues depending on the alpha chains comprising the collagen of interest [50]. In the case of a hydroxyproline standard, mass ratios of Hyp to collagen of 1∶7.5–12.5 have been used [51], [52], though few cite the primary source from which their assumptions were derived. This ratio of hydroxyproline to collagen may not be exactly the same for native and engineered tissues. For this reason, it is now apparent that a hydroxyproline to collagen ratio for engineered constructs must be determined. Additionally, matrix organization and collagen crosslinking may be contributing to mechanical properties of self-assembled costochondral cell constructs [46]. Future efforts will also seek to elucidate the potential role of matrix organization, via SEM and TEM, and pyridinoline crosslinks, via high performance liquid chromatography, in mechanical properties of self-assembled costochondral cell neocartilage.

### Conclusions

This study showed that expansion medium supplementation modulates matrix synthesis and mechanical properties of self-assembled costochondral cell neocartilage. In serum-free chondrogenic medium, TFP supplementation enhanced the ability of costochondral cells to generate biochemically and biomechanically robust neocartilage upon 3D construct formation using the self-assembly process. Conversely, bFGF demonstrated a surprisingly poor chondrogenic effect during costochondral cell expansion. However, the increased type I/II collagen ratio and decreased GAG content in the constructs expanded with FBS or bFGF alone, demonstrated that fibrous cartilage may also be developed from a single costochondral cell source. As such, costal cartilage provides a clinically relevant cell source generating a range of hyaline to fibrous cartilage upon self-assembly.

Additionally, this study demonstrated that lower construct seeding density improves matrix synthesis and mechanical properties of self-assembled costochondral cell neocartilage. The Low seeding density (2×10^6^ cells per 5 mm dia. construct) increased matrix content and mechanical properties, in all assays performed. Neocartilage engineered in this work is reflective of native articular cartilage, including both hyaline and fibrous cartilages. The interaction between Low seeding density and TFP supplementation during expansion resulted in constructs possessing GAG content and compressive moduli in the range of native TMJ condylar cartilage.
